# The Influence of Long-Time Storage on the Structure and Properties of Multi-Block Thermoplastic Polyurethanes Based on Poly(butylene adipate) Diol and Polycaprolactone Diol

**DOI:** 10.3390/ma16020818

**Published:** 2023-01-14

**Authors:** Marina A. Gorbunova, Denis V. Anokhin, Ainur F. Abukaev, Dimitri A. Ivanov

**Affiliations:** Laboratory of Structural Methods of Materials Investigation, National University of Science and Technology MISIS, Leninskiy Prospekt 4s1, 119049 Moscow, Russia

**Keywords:** thermoplastic polyurethane, shape memory materials, long-time crystallization, polycaprolactone, poly(butylene adipate)

## Abstract

A series of semi-crystalline multi-block thermoplastic polyurethanes (TPU), containing poly(butylene adipate) (PBA), polycaprolactone (PCL) and their equimolar mixture (PBA/PCL) as a soft segment was synthesized. The changes in the physical-mechanical and thermal properties of the materials observed in the course of a 36-month storage at room temperature were related to the corresponding structural evolution. The latter was monitored using Fourier transform infrared spectroscopy (FTIR), differential scanning calorimetry (DSC), wide-angle X-ray diffraction (WAXS) and mechanical tests (tensile strength test). The effects of the composition of the soft segment on the phase separation and crystallization of the soft segment were analyzed in detail. It was found that the melting temperature of the crystalline phase increases with storage time, which is associated with hindering of the phase separation of the hard and soft segments of the TPU samples as it was detected by FTIR.

## 1. Introduction

Semi-crystalline thermoplastic polyurethanes (TPU) are materials with outstanding structural flexibility giving rise to a wide variety of applications from biomaterials [1,2] to additive manufacturing [3]. The elastomeric nature derives from its blocky structure where polymer chains are composed of alternating sequences of soft and hard segments. The soft segments are generally amorphous or crystallizable polyesters such as poly(butylene adipate) (PBA) [3], polycaprolactone (PCL) [4] and their mixtures [5,6], poly-l-lactide [7], poly(1,10-decylene adipate) [8], polycarbonate [9], polypropylene glycol [10], poly(tetramethylene ether) [10], polyhexamethylene carbonate [11], etc., with specific thermal, mechanical and biodegradable properties. The urethane hard segments are formed by the isocyanate and the chain extender moieties. The rigidity of the polymer is determined by the density of hydrogen bonds between hard segments, which play a role of physical crosslinks in soft polyester matrix [9]. The soft crystallizable blocks provide TPU with shape memory effect (SME), that is, the ability to restore its original shape upon exposure to an external stimulus, most often being temperature. 

It was shown in our previous publications that variation of the soft block composition defines resulting mechanical properties of thermoplastic polyurethane-urea [1]. The mechanical modulus, tensile strength and elongation at break can vary in a wide range due to variation of crystalline phase morphology. The thickness of crystallites and total degree of crystallinity of the soft block in this case are connected with polyurethane surrounding that form a phase-separated structure. For PBA-based TPUs the crystallization kinetics and crystal phase composition was analyzed as a result of competition between the nucleation and crystal phase growth, from one side, and phase separation efficiency above the melting point of PBA and linear, aromatic and cycloaliphatic urethane blocks, from the other side. In general, for TPU the phase separation of hard and soft blocks that governs the final morphology, mechanical and thermal properties [12,13]. The incompatibility between the blocks can be finetuned not only by changing the composition and ratio of soft and hard segments but also by variation of hydrogen bonding caused by addition of nanofillers or by use of specific thermal programs [14,15,16,17,18,19,20,21]. Estimation of the hydrogen bonding density, for example by IR-spectroscopy, can be a good tool for the prediction of mechanical behavior of the materials. 

The phase separation also characterizes physical aging, e.g., change of mechanical, thermodynamic and other properties of materials during long-time storage at room temperature. The increase in the degree of crystallinity and melting point is observed for semi-crystalline polymers because of recrystallization and improvement of crystal phase quality. It was noticed that crystalline regions affect chain mobility in non-crystalline regions resulting in increase in the glass transition temperature [22]. Crystallites form an additional physical network and provide higher values of Young’s modulus in such materials. In our previous work, we found three types of supramolecular organization, which define the behavior of semi-crystalline polyurethane-urea materials under heating and uniaxial deformation [23]: a phase-separated amorphous state (1) obtained immediately after heating above the melting point of soft segment which transforms to semi-crystalline morphology (2) upon several hours of crystallization. During stretching, the isotropic semi-crystalline morphology (2) evolves into fibrillar structure (3) with a high strength. The latter can be returned back to state (1) by heating above the melting point of PBA. Long storage of morphology (2) results in slow post-crystallization and ordering of crystalline phase over several months and in ultraslow phase separation of blocks over several years. The effect of crystallization on the mechanical properties of materials was found to be complex. On the one hand, crystallites form an additional physical network. On the other hand, the crystallization of polyester hinders the phase separation. The competition between the formation of crystalline phase and the growth of phase-separated domains defines multi-step changes in mechanical and thermal properties of the samples during long-time storage. 

Since the physical aging affects the mechanical and thermal properties of the TPUs, slowing down of the process will allow the TPU-based products to be used for longer periods of time. However, this problem has not been described in the literature in detail yet. The present work is aimed at studies of the evolution of the structure and properties of TPU on the time-scale from one day to several years. 

To study the role of the phase separation kinetics on the physical aging, a series of semi-crystalline multi-block TPUs containing PBA, PCL and/or their equimolar mixture (PBA/PCL) as a soft segment were synthesized by the three-reactor method. The effect of the composition of soft segments on the microstructure and crystallization behavior of the TPUs were investigated by Fourier transform infrared spectroscopy (FTIR), wide-angle X-ray diffraction (WAXS) and differential scanning calorimetry (DSC). The details of a multi-step crystallization and phase separation of semi-crystalline TPUs with one or two crystallizable soft segments have been investigated. The obtained results help to predict the mechanical and thermal characteristics of samples during their natural aging and contribute to the production of materials with controllable properties.

## 2. Materials and Methods

### 2.1. Materials

Poly(butylene adipate) diol (PBA) (Huakai Resin Co., Ltd., Jining, China, M_n_ = 2000 Da) and polycaprolactone diol (PCL) (Merck, Darmstadt, Germany, M_n_ = 2000 Da) were vacuum-dried for 4 h at 80 °C before use. The OH-groups content determined by chemical method [24] was 1.7 wt%. 2,4-toluene diisocyanate (TDI) and 1,6-hexamethylene diisocyanate (HMDI)) (Merck, Darmstadt, Germany) were distilled in vacuum at 50–55 °C/12 mm Hg and stored in sealed ampoules. Additionally, 1,4-butanediol (BD) (Merck, Darmstadt, Germany) was distilled over freshly powdered calcium hydride under reduced pressure. Catalyst dibutyltin dilaurate was purchased (Merck, Darmstadt, Germany) and used as received.

### 2.2. Synthesis of Multi-Block Thermoplastic Polyurethane (TPU)

Synthesis of multi-block thermoplastic polyurethane (TPU) was carried out by the three-stage method developed by us earlier [1,25]. Two crystallizing diols with similar molecular weight 2000 Da were used in different weight ratios PBA/PCL (in %) 100/0 for TPU-381(PBA); 0/100 for TPU-382(PCL) and 50/50 for TPU-383(PBA/PCL). Urethane-diol fragments based on aliphatic and aromatic diisocyanates and chain extender BD form the hard segments. 

**Scheme 1:**
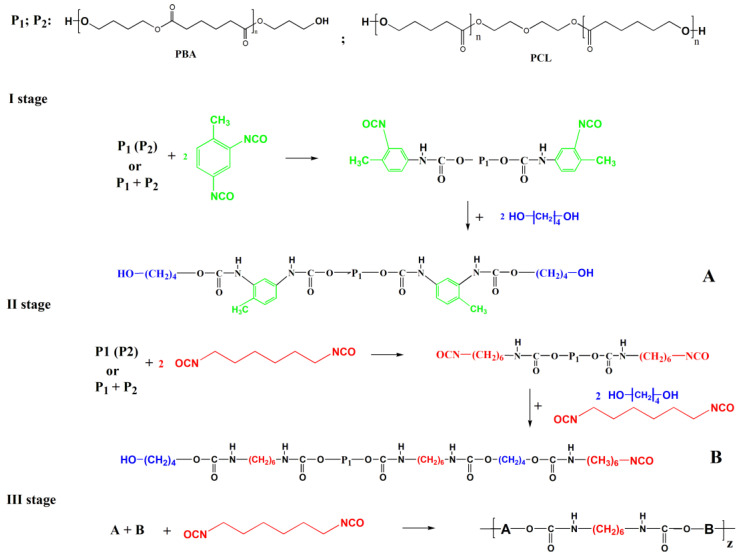
General scheme of synthesis of TPUs. (**A**) I stage—synthes of macrodiol, (**B**) II stage—synthes of macroisocyanate.

The synthesis methodology presented in this paper differs from that developed earlier [1] in that the synthesis was performed without an amine chain extender to decrease the hardness of the materials and the diol mixture was in each of the two reactors. In the first reactor of TPU synthesis, a macrodiol from diol (PBA, PCL, and a mixture of them) and TDI in the presence of BD was prepared (I stage). A macrodiol from diol (PBA, PCL, and a mixture of them), HMDI and BD were synthesized in the second reactor (II stage). Then, the reaction products from two reactors were mixed and linked by addition of HMDI in the stoichiometric ratio [NCO]/[OH] = 1 in a 3rd reactor (III stage). As a result, three polymers have been synthesized, where PCL and PBA are linked in different combinations with TDI and a more flexible HMDI (Table 1). Upon reaching the degree of conversion of NCO groups of~98%, the reaction mass was poured into a flat Teflon container and dried at 40 °C during a day until constant weight. The control of the full course of the reaction was carried out by IR spectra on the complete disappearance of absorption bands of isocyanate (*ν*_NCO_ = 2272 cm^−1^) and hydroxyl groups (*ν*_OH_ = 3623 cm^−1^) (Figure S1a). The molecular mass distribution of the TPUs was measured by gel permeation chromatography (GPC) using Waters GPCV 2000 chromatograph equipped with refractometric and viscosimetric detectors. The measurements were performed at the temperature of 35 °C. All samples were dissolved in tetrahydrofuran (THF). Polystyrene standards were used to make the calibration curve. Molecular weights and polydispersity of all polymers are given in Figure S2 and Table S1 of Supplementary Materials. The scheme of synthesis of TPU is presented in Scheme 1.

The hard segment mass fraction for all samples is 31%. The hard segment molar ratio (HS) was defined as follows:

$$\mathrm{HS}\left(\%\right)=\frac{\left(1+n\right)M_{\mathrm{TDI}+\mathrm{HMDI}}+nM_{\mathrm{BD}}}{\left(1+n\right)M_{\mathrm{TDI}+\mathrm{HMDI}}+nM_{\mathrm{BD}}+M_{\mathrm{diol}}}*100\%,$$

where *M*_diol_, *M*_TDI+HMDI_, *M*_BD_ are the molecular weights of PBA and/or PCL, diisocyanates and 1,4-butanediol, respectively; *n* is the moles of BD.

Table 1 shows the composition and ratio of components in the synthesized TPUs. In the following, the samples are noted as TPU-381(PBA)-L, where L is storage time in months. Over the storage the samples were placed in plastic boxes and kept in darkness at normal conditions.

### 2.3. Characterization

For identification of the obtained polymers and analysis of hydrogen bonding the FTIR spectroscopy was used. The spectra were recorded on a Brucker Alpha spectrometer using a multiple attenuated total reflection (ATR) module under the following conditions: measurement range 4000–500 cm^−1^, measurement step 2 cm^−1^, and the number of scans per spectrum-56. The absence of absorption bands for *υ*_NCO_ at 2272 cm^−1^ and *υ*_OH_ at 3623 cm^−1^ indicates the completeness of the reaction.

The thermal properties were analyzed by differential scanning calorimetry (DSC) using a DSC 30 (Mettler Toledo) calorimeter at a heating/cooling rate of 10 °C/min under nitrogen atmosphere. Glass transition temperature was measured during the second heating scan from −80 to 100 °C. The temperature and enthalpy of melting was calculated from the first scans from 23 to 100 °C. In case of multiple endothermic events each melting peak was fitted with Gaussian function. The Δ*H* and *T*_max_ were calculated as area and maximum of corresponding Gaussian peak, respectively. *T*_onset_ is defined as intersection of tangent line at halfwidth point with *x*-axis (see Figure S3 for details). The degree of crystallinity ($\chi_{c}$) of PBA and PCL blocks was determined by Equation (1):

(1) $$\chi_{c}=\frac{\Delta H_{m}}{\Delta H{{}^{\circ}}_{m}}\;$$

where Δ*H_m_*—total melting enthalpy of PBA or PCL measured from DSC curves, Δ*H*°*_m_* is melting enthalpy of 100% crystalline PBA (95 J/g) and PCL (203 J/g), respectively. These values were calculated previously from DSC and X-ray diffraction data of the as-received polyols [26].

Crystallization kinetics of the TPUs were performed under isothermal conditions. A sample (10 ± 0.2 mg) was heated above the melting point of the soft segment (100 °C) and then quickly cooled to the crystallization temperature of 25 °C. The crystallization process was monitored by appearance of exothermic crystallization peak on a time-resolved DSC curve. A change in the degree of crystallinity *χ*(*t*) during the isothermal crystallization of PBA was determined from the ratio between the enthalpy of melting of the crystalline phase of the sample and the enthalpy of melting corresponding to 100% degree of crystallinity of polyols according to Equation (2):

(2) $$\chi \left(t\right)=\frac{\int_{o}^{t}\left(\frac{dH}{d\tau }\right)d\tau }{\Delta H{{}^{\circ}}_{m}\mathrm{PBA}}$$

where *dH*/*dτ* is the heat flow during crystallization. 

The effect of the phase separation processes on the crystallization kinetics in the isothermal regime was explored in more detail with the classical Avrami equation [27,28] and simplified by Meares [29] and Hay [30]:

(3) $$1-\chi \left(t\right)=\exp(-Kt^{n})$$

where *χ(t)* is the degree of crystallinity at time *t*, the exponential factor n is the so-called the Avrami constant which depends on the mechanism of nucleation and shape (geometry) of the growing crystals, and parameter *K* is the rate constant including the rates of nucleation and crystal growth. To estimate the parameters *n* and *K*, the experimental dependence *χ(t)* is represented in double logarithmic coordinates:

(4) log(−ln(1−χ(t))=nlogt+logK

Using the values of *n* and *K*, one can calculate the half-time value (*t*_50%_) for the crystallization:

(5) $$t_{50\%}={\left(\frac{\ln2}{K}\right)}^{1/n}\;$$

Wide-angle X-ray diffraction analysis (WAXS) of samples was performed using a diffractometer with a 1.54 Å wavelength equipped with a 2D Dectris EIGER2 S 1M detector. The sample-to-detector distance was 47 mm, exposure time was 1 h. The modulus of the scattering vector ***s*** (|***s***| = 2 sin*ϑ/λ*, where *ϑ* is the Bragg angle, *λ* is the wavelength and |***s***| is the norm of the ***s***-vector) was calibrated using several diffraction orders of lanthanum hexaboride. The measurements were carried out in vacuum. The analysis of X-ray data, including background subtraction and the radial integration of 2D diffractograms, was performed in a home-built software environment.

Mechanical tests (tensile strength test) were performed on films after heating to 110 °C and storage at room temperature. The tests were performed on a Zwick TC-FR010TH Material Testing Machine at 50 mm/min stretching rate.

## 3. Results

### 3.1. Tensile Behavior of TPUs at Long-Time Storage

**Figure 1:**
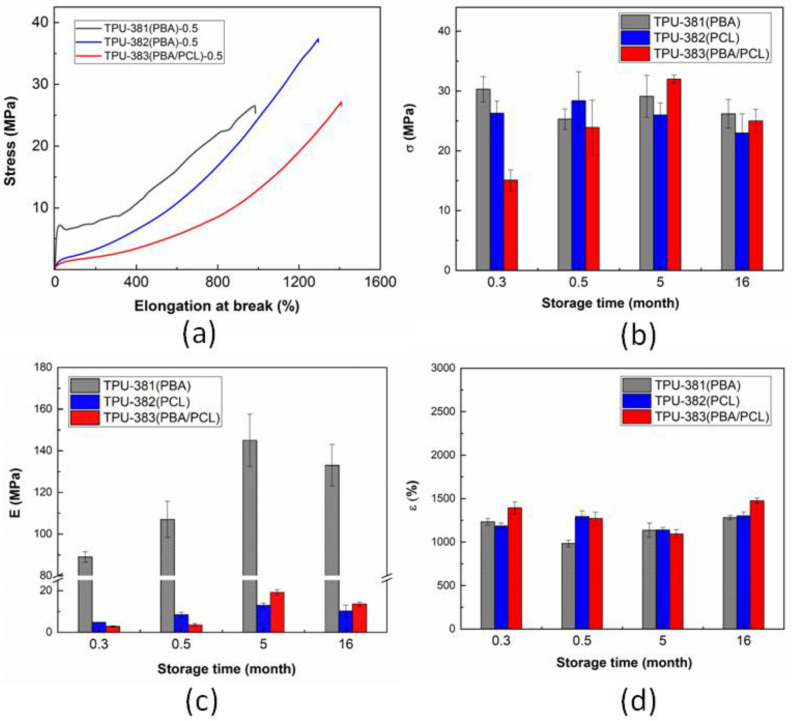
Stress–strain curves of the studied TPUs (**a**) and tensile strength (**b**), Young’s modulus (**c**), and elongation at break (**d**) as a function of storage time for TPUs with different compositions of soft segments.

The stress–strain curves of TPUs after 2 weeks of storage are shown in Figure 1a. It can be seen that TPU-381(PBA)-0.5 shows the mechanical behavior typical of rigid thermoplastics with high Young’s modulus (Figure 1a, black curve) [31]. In contrast, TPU-382(PCL)-0.5 and TPU-383(PBA/PCL)-0.5 samples demonstrate the stress–strain curve of a typical elastomer with low Young’s modulus (E) and the highest elongation at break (ε) (Figure 1a, blue and red curves). This clearly indicates that the type of polyol is important in the design of SME polymers. 

As mentioned above, the SME is related to the formation of the crystalline phase of soft segment that demonstrates fast crystallization from amorphous state and slow aging of the material for months and even years [23]. The Figure 1b–d shows variation of Young’s modulus (E), elongation at break (ε) and tensile strength (σ) during 16 months. For TPU-381(PBA) E increases from 89 to 145 MPa after five months of storage and then stay unchanged (Figure 1c, gray bars). On the contrary, σ and ε (Figure 1b, gray bars; Figure 1d, gray bars) of TPU remain constant during storage (28 MPa and 1160%, respectively). An increase in the Young’s modulus without a significant change in strength and elasticity is probably associated with slow phase separation of hard and soft blocks and corresponding change in the amorphous phase composition [32]. For elastomer TPU-382(PCL) we detect hardening of the samples due to slow crystallization of the initially amorphous soft blocks. As a result, Young’s modulus increases from 4.8 to 51.2 MPa without significant variation in ε and σ (Figure 1b–d, blue bars). For TPU-383(PBA/PCL) the hardening process is less pronounced because of slower crystallization. However, in addition to increase of modulus from 2.8 to 13.6 MPa (Figure 1c, red bars), the growth of strength (from 15.1 to 25.0 MPa, Figure 1b, red bars) without a significant change in elasticity (Figure 1d, red bars) was found. A certain decrease in elongation at break and increase in elastic modulus after 16 month of storage can be explained by improvement of phase separation of soft and hard domains (Table S2).The variation of the σ and ε can be associated with formation of nanodomains of hard and soft blocks [33]. The process of phase separation during long-term storage is the most pronounced for TPU-383(PBA/PCL) films due to low crystallization rate of both PBA and PCL blocks. It should be mentioned that the found hardening is not related to chemical cross-linking of the polymer chains (see Figure S1). The detailed results of mechanical tests (tensile strength test) are presented in Table S2. 

### 3.2. Thermal Properties of TPUs at Long-Time Storage

The analysis of soft block crystallization during storage was performed by DSC. The analysis shows that for TPU-381(PBA) the glass transition temperature does not change during aging, which indicates the absence of formation or destruction of physical cross-links due to phase separation. For TPU-382(PCL) the glass transition temperature increases insignificantly during storage. On the contrary, for TPU-383 a significant increase in the glass transition temperature is observed during storage, indicating large changes in the amorphous phase. 

**Figure 2:**
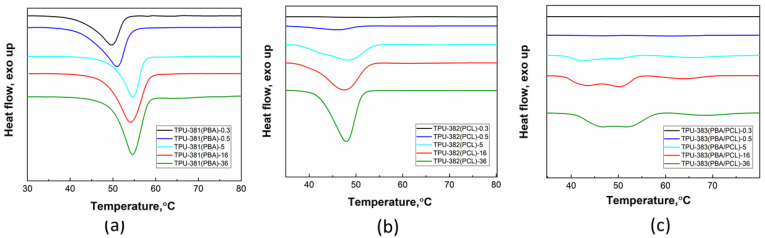
DSC curves for (**a**) TPU-381(PBA), (**b**) TPU-382(PCL) and (**c**) TPU-383(PBA/PCL) given for samples with different storage times in months.

The reversibility of thermal processes in TPU samples with different storage period was studied from analysis of the first heating scans from room temperature to 100 °C above PBA and PCL melting point. As can be seen on the first scan of fresh TPU-381(PBA)-0.3 sample the endothermic melting peak of the PBA segment is observed with onset at 43 °C (Figure 2a). In storage process the onset of the peak shifts to 50 °C with moderate change in degree of crystallinity after 36 months of storage (Figure 2a, green line). Such a trend can be explained by fast crystallization of PBA within the first few hours after film preparation. Therefore, the physical aging of this sample is related to perfectioning of the crystalline phase with strongly hindered chain mobility. The DSC scans TPU-382(PCL) reveals appearance of endothermic melting peak with onset at 37 °C in only two weeks after film preparation (Figure 2b, black and blue curves). During storage, the onset shifts to 42 °C with gradual growth of the melting enthalpy (Figure 2b, blue line). In three years the degree of crystallinity reaches 19% imparting good thermoplastic properties to the polymer as detected by mechanical tests (tensile strength test) (Table 2). Consequently, for TPU-382(PCL) crystallization of PCL occurs in parallel with improvement in crystal packing of the previously formed crystalline domains. The most interesting behavior demonstrates TPU-383(PBA/PCL) containing two crystallizable blocks (Figure 2c). Two weak endothermic peaks appear on DSC scans of TPU-383(PBA/PCL)-0.5 which can be attributed to melting of PCL (onset at 40 °C) and PBA (onset at 53 °C) crystals (Figure 2c, blue curve). With storage time, the onsets stay almost unchanged with monotonic growth of the melting enthalpy of both peaks, but after 5 months the third melting event appears at 57 °C (Figure 2c, light blue curve). The latter peak we explain as being due to population of regular thick PBA crystals formed in amorphous nanodomains of the soft block. Earlier, we have observed similar complex thermal behavior during long-term storage for semi-crystalline polyurethane-urea systems [23]. Interestingly, in contrast to TPU-381(PBA) and TPU-382(PCL) the crystallization rates of PBA and PCL become comparable due to slow phase separation of the blocks in the initial state [25]. Parallel formation of two types of crystallites slows crystallization of PBA but from on the other side accelerates phase separation because of high chain mobility of the amorphous polymer. In this process, more perfect PBA crystals can be formed. However, mutual hindering of crystal nucleation leads to a decrease in the crystallization rate and total degree of crystallinity (Table 2).

### 3.3. Isothermal Crystallization Kinetics of TPUs 

**Figure 3:**
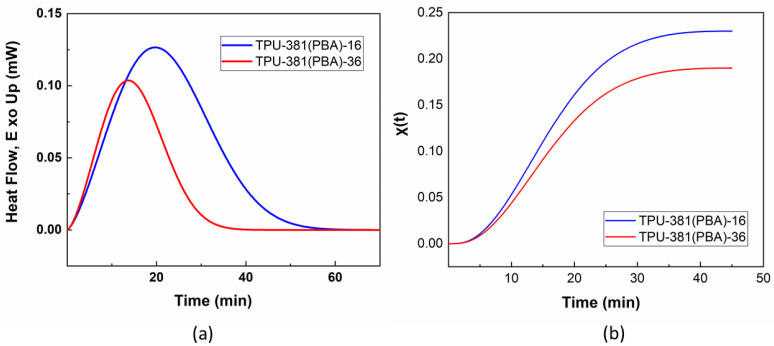
Isothermal crystallization exotherms (**a**) and curves χ(t). (**b**) of TPU-381(PBA)-16 (blue line) and TPU-381(PBA)-16 (red line) taken at 25 °C.

As we have shown earlier, slow phase separation process can be detected by a change in recrystallization kinetics when larger domains of the soft block promotes faster nucleation and higher crystal growth rate [23]. Figure 3 shows crystallization exotherms of TPU-381(PBA) films at 25 °C as a function of storage time. One can see that from 16 to 36 months the physical aging results in a drop of crystallization half-time t_50%_ from 22.5 to 15.9 min (Figure 3a). The corresponding decrease of degree of crystallinity from 23 to 19% is explained by formation of less regular and/or smaller crystals during fast crystallization process.

The normalized dependences χ(t) were plotted in Avrami coordinates log[−ln(1 − χ(t)] vs. log(*t*) to calculate *n* and *K*, which are governed by the nucleation kinetics and shape of the growing crystals. The analysis reveals that the Avrami parameters do not change significantly with storage time being equal *n* = 2.4 ± 0.1, *K* = 59 ± 19 min^−1^, respectively. The found value of *n* is typical of 3D spherulitic growth with thermal (homogenous) nucleation type with diffusion limitation [33].

### 3.4. Crystal Structure and Phase Composition of TPUs 

**Figure 4:**
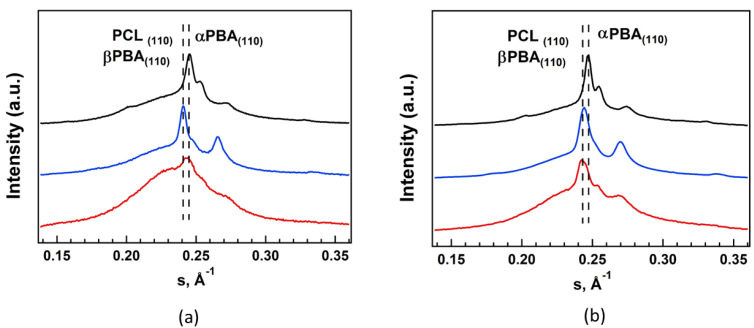
Diffractograms of TPU-381(PBA) (black curves), TPU-382(PCL) (blue curves) and TPU-383(PBA/PCL) (red curves) after 2 weeks (**a**) and 36 months (**b**) of storage.

Composition of crystal phase of the soft block was studied by WAXS (Figure 4). Diffraction profiles of both fresh TPU-381(PBA)-0.5 and old TPU-381(PBA)-36 reveal reflections at *s* = 0.245 Å^−1^ (d = 4.09 Å) of crystalline α-phase of PBA (Figure 4a,b, black curves) [34]. WAXS curves of TPU-382(PCL) show intense peaks at *s* = 0.241 Å^−1^ (*d* = 4.15 Å) of orthorhombic crystal phase of PCL [35] (Figure 4a,b, blue curves). Diffractogram of TPU-383(PBA/PCL)-0.5 demonstrates two broad overlapped peaks in the range from 0.240 to 0.247 Å^−1^ (Figure 4a,b, red curves) indicating formation of mixture of PCL and PBA phases. Since DSC data show very low concentration (less than 1%) of PCL crystal phase after 2 weeks of storage (see Table 2), we suppose presence in TPU-383(PBA/PCL)-0.5 of monoclinic β-modification of PBA which also has the most intense peak at *s* = 0.241 Å^−1^ [36]. For PBA homopolymers this phase is considered as metastable and transforms to stable α-modification during heating to 31–35°C or during long storage at room temperature [36]. However, in multi-block polyurethanes β-crystals of PBA can exist for month in geometrically confined nanodomains [37]. After storage for 36 months the content of PCL crystalline phase increases to 6% but relative intensity of the reflections at 0.241 and 0.245 Å^−1^ stay approximately the same. We expect that the decrease in relative content of β-phase of PBA is related to formation of amorphous domains of PBA that stimulates crystallization in thermodynamically stable α-phase. Unfortunately, it is impossible to identify directly the content of β-phase of PBA, because it is very close to physical parameters to crystal phase of PCL.

### 3.5. Microphase Structure and Hydrogen Bonding of TPUs

As was mentioned above the effectiveness of phase separation can be estimated from density of hydrogen bonding between urethane blocks. 

**Figure 5:**
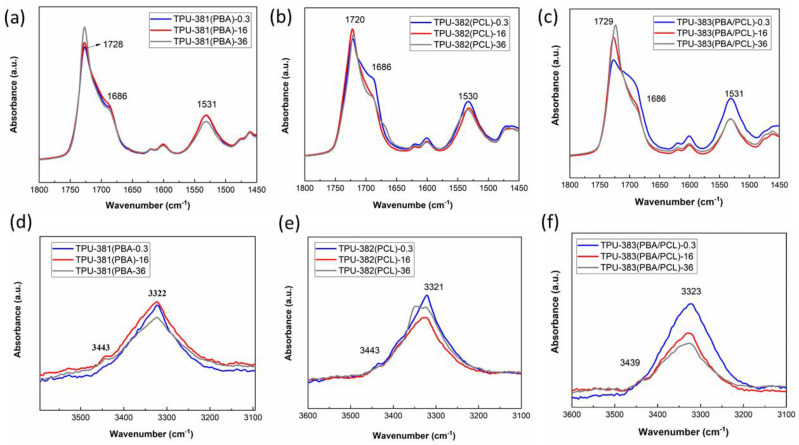
Infrared spectra of the TPU-381(PBA) (**a**,**d**), TPU-382(PCL) (**b**,**e**) and TPU-383(PBA/PCL) (**c**,**f**) in the region of stretching vibrations of carbonyl (**a**–**c**), amide groups (**d**–**f**).

In the FTIR spectra of all samples (Figure 5) one can see absorption bands corresponding to stretching and bending vibrations of the -NH group (*υ*_NH_~3500–3200 cm^−1^ and δ_NH_~1580–1490 cm^−1^) and stretching vibrations of the carbonyl group (*υ*_C=O_~1800–1640 cm^−1^), which characterize the formation of urethane bonds (Figure S1). The presence of the corresponding bands in the FTIR spectra indicates the formation of inter-urethane bonds in phase-separated morphology of TPU [25,38]. The peaks at 2865 cm^−1^ and 2939 cm^−1^ are characteristic of CH_2_ groups of the soft segment (Figure S1). In the absorption region of valence vibrations of amide groups for all samples (Figure 5), a low intensity peak at 3343 cm^−1^ corresponds to free NH-groups. This indicates that most of the amide groups of hard segment are hydrogen-bonded.

The effect of the soft block composition on phase separation can be identified from analysis of FTIR spectra in the range of valence vibrations of amide and carbonyl groups. TPU-381(PBA)-0.3 exhibits two characteristic infrared bands (Figure 5a,d): the first is associated with vibrations of the hydrogen-bonded carbonyl group of ordered urethane-urethane hard segment (HS-HS) at 1686 cm^−1^, and the other is carbonyl from free or weakly associated ester and urethane groups at 1728 cm^−1^. Both bands also contain a «mixture» of free (SS,HS) and hydrogen-bonded carbonyl groups (HS-HS,HS-SS) [39]. Storage for 36 months results in variation in phase-separated morphology which was detected as change in intensity (increase for band at 1728 cm^−1^ and decrease for bands at 1686, 3322 and 1531 cm^−1^) and shift of absorption bands of amide and carbonyl groups (Figure 5a,d). 

To better describe phase-separation in studied samples after storage, we identified the positions of the bands of the urethane and ester carbonyl groups [39] in the region of the stretching vibration 1800–1680 cm^−1^, which for TPU-381(PBA)-0.3 indicates the presence of five spectral bands of interest: 1686 (HS-HS), 1700 (HS-HS), 1708 (HS-SS), 1728 (SS-SS) and 1740 (SS) cm^−1^ (Figure S4a, Table S3). For stored sample TPU-381(PBA)-36, the second derivatives show a certain decrease in bands of the mixed phase (HS-SS) and increase in band of PBA crystal phase (SS-SS) at 1728 cm^−1^ due to improvement in phase separation of hard and soft blocks (Figure 5a, Figure S4a gray curve, Table S3). 

For TPU-382(PCL)-0.3 the second derivatives allow to select the following absorption bands: 1686, 1708, 1721, 1732 and 1740 cm^−1^ (Figure S4b, Table S3). On the one hand, high crystallization rate of PBA and efficient phase separation compared to PCL segment results in the presence of large amount of dipole-dipole interactions of carbonyl groups in the PBA crystal phase and decrease in the soft fragment mobility [40]. On the other hand, low crystallization rate of PCL at room temperature cannot stimulate phase separation which is indicated in increase in concentration of hydrogen bonds between the carbonyl group of the soft segment and NH-group of hard urethane segment (HS-SS) (Table S3). For the TPU-382(PCL)-36 we see an increase in intensity of peaks at 1720 (SS-SS) and 1686 (HS-SS) cm^−1^ and corresponding decrease in band at 1708 (HS-SS) cm^−1^ (gray curve in Figure 5b,e). Probably, slow crystallization is accompanied with phase separation of disordered blocks due to decrease in chain mobility. 

For the fresh sample TPU-383(PBA/PCL)-0.3 containing a mixture of two polyols the following bands are identified: low-intense peak of carbonyl stretching at 1729 cm^−1^, wide shoulder at 1686 cm^−1^, intense bands of amide groups at 3323 and 1531 cm^−1^ indicating high content of the mixed phase (HS-SS) formed by amide groups of urethane and carbonyl groups of esters (Figure 5c,f). From the second derivatives the following bands are selected: 1745, 1738, 1729, 1721, 1708 1686 cm^−1^ (Figure S4c, Table S3). The intense band at 1729 cm^−1^ (Figure S4c, blue curve) and weak peak at 1721 cm^−1^, typical for dipole-dipole interactions of PBA and PCL crystals (SS-SS) confirms the conclusion that PBA crystallizes before PCL. After storage for 36 month a decrease in intensity of bands at 1738 (HS), 1708 (HS-SS) cm^−1^ and growth of intensity of band at 1721 (SS-SS) cm^−1^ with decrease in bands of amide groups indicates decrease in the mixed phase (HS-SS) content and free carbonyl groups of urethane because of development of phase separated morphology and slow crystallization of PCL block. These results are in good agreement with DSC and WAXS data. More detailed analysis of phase mixing and separation will be presented in incoming publications using model polymers.

## 4. Conclusions

In the presented work, the process of physical aging of thermoplastic polyurethanes during long-time storage at room temperature was studied as a function of composition of crystallizable soft block. Based on experimental data collected for three years of the samples storage, a direct correlation between crystallization rate and effectiveness of microphase separation of hard and soft blocks was discovered. Sample TPU-381(PBA) with the highest crystallization rate of the soft block demonstrates mechanical properties typical of a thermoplastic due to relatively high content of crystalline phase formed in a few days upon sample preparation It is expected that PBA crystallization occurs directly from isotropic mixed state of hard and soft blocks. During long-term storage the initially formed morphology slowly evolves as local perfecting of crystal phase. Further phase separation and enhancement of crystallinity is limited by low mobility of chains restricted in crystalline domains of PBA and hydrogen-bonded urethane domains. Films of TPU-382(PCL) demonstrate continuous increase in crystallinity of the PCL block during storage for 16 months accompanied by perfectioning of the PCL crystal structure. Because of lower tendency for phase separation of PCL and urethane blocks it is expected that polyester also crystallizes from isotropic state as in the case of TPU-381(PBA). As a result, this sample demonstrates the strongest effect of physical aging with increase in mechanical modulus by a factor of ten providing optimal thermoelastoplastic properties. The TPU-383(PBA/PCL) sample reveals an interesting behavior with two crystallizable blocks which mutually hinder ordering of each other immediately after sample preparation. Then, during storage formation of two crystal populations of PCL and PBA takes place with compatible rates. The presence of PBA crystalline fraction with increased melting temperature and existence of metastable β-crystalline modification of PBA prompts us to assuming that in this case crystallization occurs in nanodomains of the phase-separated morphology. Comparison of FTIR spectra and their second derivatives for the studied samples at different stages of physical aging displays improvement of the phase separation in the following order: TPU-381(PBA)-36 ≤ TPU-382(PCL)-36 < TPU-383(PBA/PCL)-36. Thus, the use of various polyols with different crystallization rates and their combinations makes it possible to obtain materials with wide range of mechanical properties: from soft elastomes to rigid plastics. Moreover, the balance between crystallization of polyols and phase separation of hard and soft blocks can control evolution of the mechanical characteristics over long periods, which is important for practical use of smart SME polymers. 

## Figures and Tables

**Table 1:** Chemical composition of TPUs.

Polymer	Polymer Composition		Mass Fraction of Reagents, %			
	P1	P2	Polyol	Diisocyanate	Chain Extender	SS/HS
TPU-381(PBA)	PBA	-	69	23	8	2.2
TPU-382(PCL)	-	PCL	69	23	8	2.2
TPU-383(PBA/PCL)	PBA	PCL	69	23	8	2.2

**Table 2:** Thermal parameters for the TPUs determined from the DSC analysis.

Samples	*T_g_*, °C	*T_onset_*, °C	*T_max_*, °C	*ΔH_m_*, J/g	*χ_c_*PBA, %	*χ_c_*PCL, %
TPU-381(PBA)						
TPU-381(PBA)-0.3	−48	43	49	30	31	-
TPU-381(PBA)-0.5	−48	43	50	32	33	-
TPU-381(PBA)-5	−49	49	54	34	35	-
TPU-381(PBA)-16	−49	48	54	35	36	-
TPU-381(PBA)-36	−49	50	55	35	37	-
TPU-382(PCL)						
TPU-382(PCL)-0.3	−51	-	-	-	-	-
TPU-382(PCL)-0.5	−51	37	46	19	-	9
TPU-382(PCL)-5	−53	37	42/48	24	-	12
TPU-382(PCL)-16	−54	40	47	35	-	17
TPU-382(PCL)-36	−54	42	48	39	-	19
TPU-383(PBA/PCL)						
TPU-383(PBA/PCL)-0.3	−49	-	-	-	-	-
TPU-383(PBA/PCL)-0.5	−49	40/53/-	45/61/-	2/1/-	3	0.5
TPU-383(PBA/PCL)-5	−57	39/45/57	43/49/66	4/3/3	6	2
TPU-383(PBA/PCL)-16	−58	39/47/55	44/50/63	5/4/4	9	3
TPU-383(PBA/PCL)-36	−58	40/49/57	47/53/67	12/6/5	12	6

## Data Availability

The data presented in this study are available in article.

## Supplementary Materials

The following supporting information can be downloaded at: https://www.mdpi.com/article/10.3390/ma16020818/s1, Figure S1: Typical FTIR spectra of TPU films with different soft segment compositions after 10 days (a) and 36 (b) month of storage; Figure S2: Molar mass distribution of the synthesized TPUs; Table S1: Molecular weights and polydispersity of the synthesized TPUs; Figure S3: Example of deconvolution of DSC scan of TPU-383(PBA/PCL)-5 with multiple melting peaks: experimental curve after baseline subtraction (black), individual Gaussian peaks (blue), sum of Gaussian peaks (red), tangent lines at half-width point (green). Table S2: Mechanical properties of TPUs; Table S3: Characteristic IR bands for phase-mixed and phase-separated systems; Figure S4: Second derivatives of the IR spectra of the TPUs depending on storage period.
