# Supplementary material for: The Influence of Long-Time Storage on the Structure and Properties of Multi-Block Thermoplastic Polyurethanes Based on Poly(butylene adipate) Diol and Polycaprolactone Diol
Source: Materials (Basel). 2023 Jan 14;16(2):818. doi: 10.3390/ma16020818 (PMC9865919; doi:10.3390/ma16020818)
Supplement: Supplementary file 1 [file materials-16-00818-s001.zip › materials-2101753-supplementary.pdf]

Supplementary Materials

The Influence of Long-Time Storage on the Structure and Properties of Multi-Block Thermoplastic Polyurethanes Based on Poly(butylene adipate) Diol and Polycaprolactone Diol

Marina A. Gorbunova *, Denis V. Anokhin, Ainur F. Abukaev and Dimitri A. Ivanov

Laboratory of Structural Methods of Materials Investigation, National University of Science and Technology MISIS, Leninskiy Prospekt 4s1, 119049 Moscow, Russia
* Correspondence: mflute2008@yandex.ru; Tel.: +7-905-509-35-21

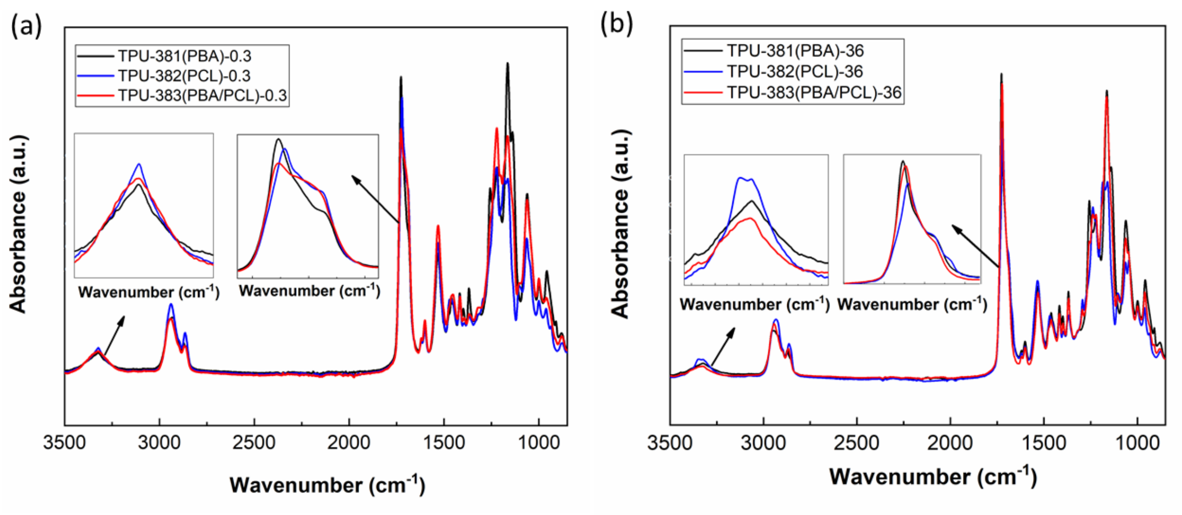

Figure S1. Typical FTIR spectra of TPU films with different soft segment compositions after 10 days (a) and 36 (b) month of storage.

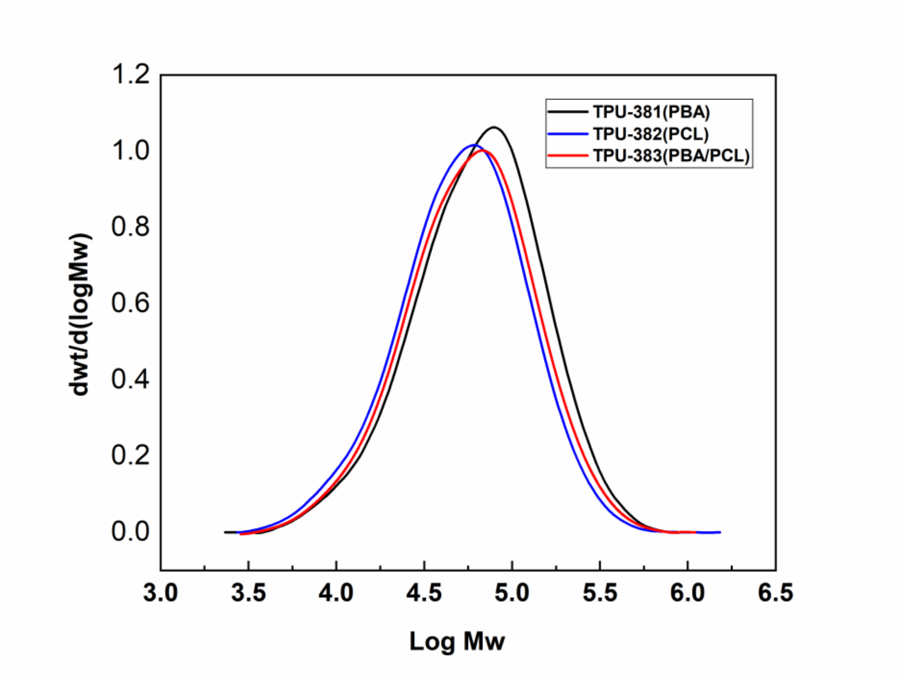

Figure S2. Molar mass distribution of the synthesized TPUs.

Table S1. Molecular weights and polydispersity of the synthesized TPUs.

Sample	Mn	Mw	Mw/ Mn
TPU-381(PBA)	42 200	89 700	2.1
TPU-382(PCL)	33 000	78 600	2.4
TPU-383(PBA/PCL)	29 800	73 000	2.4

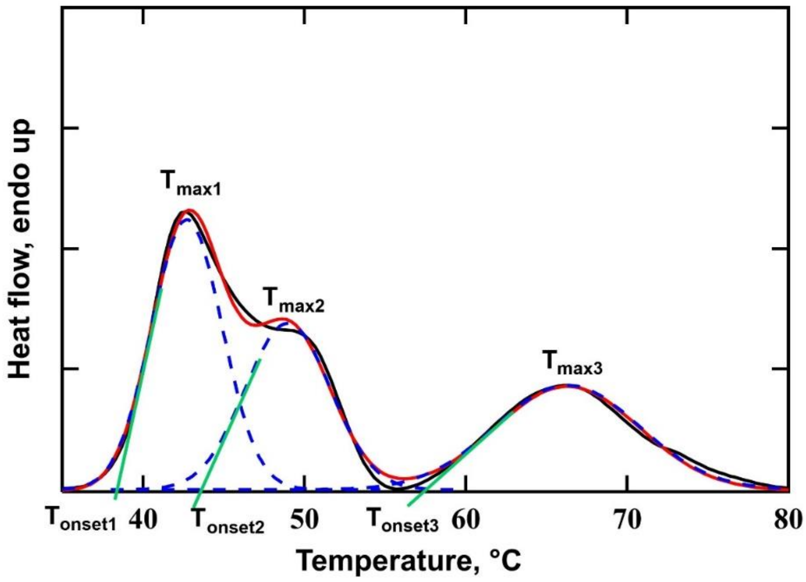

Figure S3. Example of deconvolution of DSC scan of TPU-383(PBA/PCL)-5 with multiple melting peaks: experimental curve after baseline substruction (black), individual Gaussian peaks (blue), sum of Gaussian peaks (red), tangent lines at half-width point (green).

Table S2. Mechanical properties of TPUs.

Sample	Storage time, month	E, MPa	ε,%	σ, MPa
TPU-381(PBA)-0.3	0.3	89 ± 5	1230 ± 40	30.3 ± 2.1
TPU-381(PBA)- 0.6	0.5	107 ± 9	990 ± 40	25.3 ± 1.7
TPU-381(PBA)-5	5	145 ± 13	1140 ± 80	29.1 ± 3.5
TPU-381(PBA)-16	16	133 ± 10	1280 ± 20	26.2 ± 2.4
TPU-382(PCL)-0.3	0.3	4.8 ± 0.5	1180 ± 30	26.3 ± 2.0
TPU-382(PCL)-0.6	0.5	8.4 ± 1.3	1300 ± 60	28.4 ±4 .8
TPU-382(PCL)-5	5	12.9 ± 1.2	1140 ± 30	26.0 ± 2.0
TPU-382(PCL)-16	16	51.2 ± 2.9	1300 ± 50	23.0 ± 3.2
TPU-383(PBA/PCL)-0.3	0.3	2.8 ± 0.3	1400 ± 70	15.1 ± 1.7
TPU-383(PBA/PCL)-0.6	0.5	3.6 ± 0.7	1300 ± 70	23.9 ± 4.6
TPU-383(PBA/PCL)-5	5	19.2 ± 1.2	1100 ± 50	32.0 ± 0.7
TPU-383(PBA/PCL)-16	16	13.6 ± 0.9	1476 ± 30	25.0 ± 1.0

Table S3. Characteristic IR bands for phase-mixed and phase-separated systems.

Samples	NH (free)	NH (bonded) Phase-separated	NH (bonded) Phase-separated	C=O (free) SS Polyol	C=O (free) HS Urethane	C=O SS-SS (dipol-dipol)	C=O (bonded) HS-SS Phase-mixed	C=O (bonded) HS-HS Phase-separated
	**3500-3400**	**3350-3300**	**1539-1530**	**1740-1738**	**1738-1726**	**1735-1710**	**1711-1705**	**1704-1682**
**TPU-381(PBA)**								
TPU-381(PBA)-0.3	3443	3322_{max}	1531	1740	small amplitude	1728	1708	1700, 1686
TPU-381(PBA)-16	3443	3322_{max}	1531	1740	small amplitude	1728	1708	1700, 1686
TPU-381(PBA)-36	3443	3322_{max}	1531	1740	small amplitude	1728	1708	1700, 1686
**TPU-382(PCL)**								
TPU-382(PCL)-0.3	3443	3323_{max}	1531	1740	1732	1721	1708	1700, 1686
TPU-382(PCL)-16	3443	3323_{max}	1531	1740	1732	1721	1708	1700, 1686
TPU-382(PCL)-36	3443	3348, 3322	1533	1740	1732	1720	1708	1700, 1687
**TPU-383(PBA/PCL)**								
TPU-383(PBA/PCL)-0.3	3439	3323	1531	1745	1738	1729, 1721	1708	1700, 1686
TPU-383(PBA/PCL)-16	3439	3323	1531	1745	1738	1729, 1721	1708	1700, 1686
TPU-383(PBA/PCL)-36	3439	3323	1531	1745	1738	1727, 1721	1708	1700, 1685

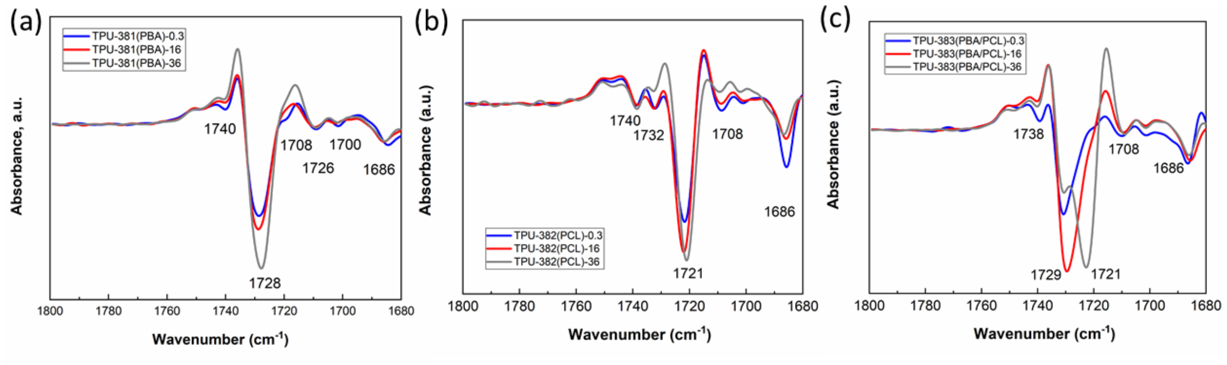

Figure S4. Second derivatives of the IR spectra of the TPUs depending on storage period.
